# Heterotrimeric Gα-subunit regulates flower and fruit development in CLAVATA signaling pathway in cucumber

**DOI:** 10.1093/hr/uhae110

**Published:** 2024-04-16

**Authors:** Lijie Han, Yafei Huang, Chuang Li, Di Tian, Daixi She, Min Li, Zhongyi Wang, Jiacai Chen, Liu Liu, Shaoyun Wang, Weiyuan Song, Liming Wang, Chaoheng Gu, Tao Wu, Jianyu Zhao, Zhaoyang Zhou, Xiaolan Zhang

**Affiliations:** Beijing Key Laboratory of Growth and Developmental Regulation for Protected Vegetable Crops, Department of Vegetable Sciences, China Agricultural University, Beijing 100193, China; Beijing Key Laboratory of Growth and Developmental Regulation for Protected Vegetable Crops, Department of Vegetable Sciences, China Agricultural University, Beijing 100193, China; Beijing Key Laboratory of Growth and Developmental Regulation for Protected Vegetable Crops, Department of Vegetable Sciences, China Agricultural University, Beijing 100193, China; Beijing Key Laboratory of Growth and Developmental Regulation for Protected Vegetable Crops, Department of Vegetable Sciences, China Agricultural University, Beijing 100193, China; Beijing Key Laboratory of Growth and Developmental Regulation for Protected Vegetable Crops, Department of Vegetable Sciences, China Agricultural University, Beijing 100193, China; Beijing Key Laboratory of Growth and Developmental Regulation for Protected Vegetable Crops, Department of Vegetable Sciences, China Agricultural University, Beijing 100193, China; Beijing Key Laboratory of Growth and Developmental Regulation for Protected Vegetable Crops, Department of Vegetable Sciences, China Agricultural University, Beijing 100193, China; Beijing Key Laboratory of Growth and Developmental Regulation for Protected Vegetable Crops, Department of Vegetable Sciences, China Agricultural University, Beijing 100193, China; Beijing Key Laboratory of Growth and Developmental Regulation for Protected Vegetable Crops, Department of Vegetable Sciences, China Agricultural University, Beijing 100193, China; Beijing Key Laboratory of Growth and Developmental Regulation for Protected Vegetable Crops, Department of Vegetable Sciences, China Agricultural University, Beijing 100193, China; Beijing Key Laboratory of Growth and Developmental Regulation for Protected Vegetable Crops, Department of Vegetable Sciences, China Agricultural University, Beijing 100193, China; Beijing Key Laboratory of Growth and Developmental Regulation for Protected Vegetable Crops, Department of Vegetable Sciences, China Agricultural University, Beijing 100193, China; Beijing Key Laboratory of Growth and Developmental Regulation for Protected Vegetable Crops, Department of Vegetable Sciences, China Agricultural University, Beijing 100193, China; College of Horticulture/Yuelu Mountain Laboratory of Hunan Province, Hunan Agricultural University, Changsha 410128, China; Beijing Key Laboratory of Growth and Developmental Regulation for Protected Vegetable Crops, Department of Vegetable Sciences, China Agricultural University, Beijing 100193, China; Beijing Key Laboratory of Growth and Developmental Regulation for Protected Vegetable Crops, Department of Vegetable Sciences, China Agricultural University, Beijing 100193, China; Beijing Key Laboratory of Growth and Developmental Regulation for Protected Vegetable Crops, Department of Vegetable Sciences, China Agricultural University, Beijing 100193, China

## Abstract

Flowers and fruits are the reproductive organs in plants and play essential roles in natural beauty and the human diet. CLAVATA (CLV) signaling has been well characterized as regulating floral organ development by modulating shoot apical meristem (SAM) size; however, the signaling molecules downstream of the CLV pathway remain largely unknown in crops. Here, we found that functional disruption of CsCLV3 peptide and its receptor CsCLV1 both resulted in flowers with extra organs and stumpy fruits in cucumber. A heterotrimeric G protein α-subunit (CsGPA1) was shown to interact with CsCLV1. *Csgpa1* mutant plants derived from gene editing displayed significantly increased floral organ numbers and shorter and wider fruits, a phenotype resembling that of *Csclv* mutants in cucumber. Moreover, the SAM size was enlarged and the longitudinal cell size of fruit was decreased in *Csgpa1* mutants. The expression of the classical stem cell regulator *WUSCHEL* (*WUS*) was elevated in the SAM, while the expression of the fruit length stimulator *CRABS CLAW* (*CRC*) was reduced in the fruit of *Csgpa1* mutants. Therefore, the Gα-subunit CsGPA1 protein interacts with CsCLV1 to inhibit floral organ numbers but promote fruit elongation, via repressing *CsWUS* expression and activating *CsCRC* transcription in cucumber. Our findings identified a new player in the CLV signaling pathway during flower and fruit development in dicots, increasing the number of target genes for precise manipulation of fruit shape during crop breeding.

## Introduction

Flowers and fruits are the reproductive organs in plants and play essential roles in natural beauty and the human diet. The *CLAVATA* (*CLV*)–*WUSCHEL* (*WUS*) signaling pathway regulates flower and fruit development by modulating shoot apical meristem (SAM) size [1]. The SAM is located at the shoot tip and is responsible for the continuous organogenesis of all aerial organs. The central zone of the SAM contains a mass of pluripotent stem cells that maintain themselves and replenish cells for organogenesis. The peripheral zone of the SAM produces leaves during the vegetative stage and flowers during the reproductive phase [2]. CLV3 is a peptide that belongs to the CLAVATA3/EMBRYO SURROUNDING REGION (ESR)-related (CLE) peptide family, secreted by stem cells and predominantly expressed in the central zone [3]. The CLV3 peptide can be perceived by a leucine-rich repeat receptor kinase, CLV1 [4, 5]. *WUS* encodes a homeodomain transcription factor that is expressed at the organization center of the SAM [6, 7]. CLV1 binding to CLV3 will result in suppression of WUS activity, whereas WUS stimulates the expression of *CLV3*, thus forming a negative feedback loop [8, 9]. *CLV2* encodes a leucine-rich repeat-like protein without a kinase domain that plays a parallel role to CLV1 during SAM regulation [10].

Mutations in the *CLV–WUS* signaling pathway genes generally lead to alteration of SAM size, changes in floral organs, and fruit development. For example, functional disruption of *CLV3*-homologous genes resulted in enlarged meristem, increased floral organ numbers, and greater fruit diameter in *Arabidopsis*, rice, and tomato [11–15]. Likewise, enhanced expression of *ZmWUS1* may cause a larger SAM in maize [16], while a gain-of-function mutation of *SlWUS* underlying the *locule number* (*lc*) locus led to fruits with more locules [17]. Therefore, functions of the *CLV3–WUS* pathway genes are widely conserved in flowering plants. Fine tuning of the *CLV3–WUS* pathway genes is of significant potential for crop breeding to produce flowers with extra organs and enlarged fruits.

Several genes have been found to function downstream of CLV signaling during SAM development and maintenance. *POLTERGEIST* (*POL*) encodes a Protein Phosphatase 2C (PP2C)that functions downstream of CLV1 receptor by transmitting signals from CLV1 to the nucleus [18]. Recent studies indicated that receptor-like cytoplasmic kinase (RLCK) PBLs can undergo phosphorylation by CLV1 to mediate *Arabidopsis* meristem size [19, 20]. Another reported downstream component of the CLV signaling pathway are heterotrimeric G proteins, which are composed of Gα, Gβ, and Gγ subunits. Mutations of G proteins result in rounded and wrinkled leaves, reduced organ size and decreased hypocotyl length in *Arabidopsis* [21–25]. Gβ was shown to interact with RECEPTOR-LIKE PROTEIN KINASE 2 instead of CLV1 to regulate SAM size in *Arabidopsis* [26]. In maize, Gα protein COMPACT PLANT2 (CT2) transmits the stem-cell-restrictive signal from CLV receptors to modulate meristem size and floral development. Mutations in *CT2* resulted in fasciated ears, increased tassel branches and spikelet density, resembling the *clv* mutant phenotypes [27]. However, whether Gα functions in the CLV signaling pathway to regulate SAM size and floral organ numbers remains unknown in dicots.

Cucumber is an important vegetable crop cultivated worldwide for over 3000 years [28]. Cucumber flowers and fruits are produced from the leaf axil, and fruits develop from the inferior ovary with three fused carpels. Cucumber fruit is generally harvested when immature (1–2 weeks after anthesis) and consumed fresh or after processing by pickling [29]. Fruit shape consists of fruit length, fruit diameter, and fruit shape index, which directly affect cucumber appearance, quality, and commercial value [30]. Here, we found a new player, a Gα-subunit in the CLV signaling pathway that regulates flower and fruit development in cucumber. We further demonstrated that the Gα-subunit interacts with CLV1 to modulate floral organ number and fruit length by inhibiting *CsWUS* expression while promoting *CsCRC* expression in cucumber.

## Results

### Functional disruption of *CsCLV3* by gene editing resulted in increased floral organ numbers and stumpy fruits in cucumber

**Figure 1:**
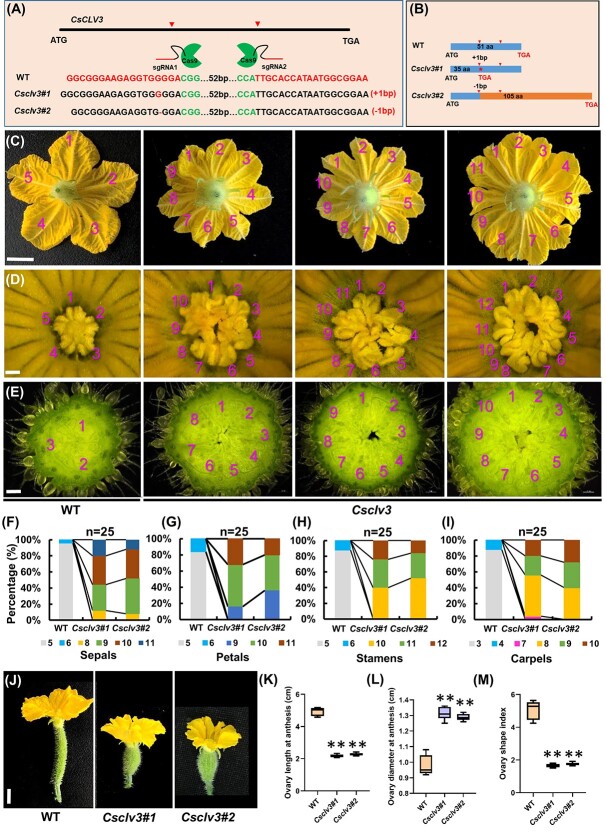
Phenotype identification of *Csclv3* mutants in cucumber. **A**, **B** Two mutation sites in *CsCLV3* generated by CRISPR/Cas9. **A** Genotype identification of *Csclv3* mutants indicated the *Csclv3#1* mutant with a 1-bp insertion and the *Csclv3#2* mutant with a 1-bp deletion, leading to premature termination of protein translation and a frameshift mutation, respectively. **B** Schematic illustration of the two mutation forms in *CsCLV3.* Red arrows represent the two target sites and the red star indicates where the protein's translation is prematurely terminated. Orange horizontal bars represent amino acids translated in *Csclv3* mutants, different from WT. **C**–**E** Representative phenotypes of petals (**C**), stamens (**D**), and carpel numbers (**E**) in *Csclv3#1* mutants and WT. The numbers in the pictures represent floral organ numbers. Scale bars = 1 cm in (**C**) and 100 μm in (**D** and **E**). **F**–**I** Quantification for percentages of sepals (**H**), petals (**I**), stamens (**J**), and carpel numbers (**I**) in WT and *Csclv3* lines (*n* = 25). **J** Representative images of cucumber ovaries at anthesis from WT and *Csclv3* mutants (scale bars, 1 cm). **K**–**M** Ovary length (**K**), ovary diameter (**L**), and ovary shape index at anthesis (**M**) in WT and *Csclv3* mutants. Values are means ± standard deviation (*n* = 8). ^**^P < 0.01 (two-tailed Student’s *t*-test).

Our previous studies using RNAi showed that the *CsWUS–CsCLV3* pathway plays an essential role in carpel number determination in cucumber [31]. Due to the intrinsic feature of instability of RNAi lines, stable *Csclv3* mutants were obtained, using the CRISPR/Cas9 system, to dissect how *CsCLV3* peptide transmits the signal during flower development. Two independent homozygous lines were chosen for further characterization. *Csclv3#1* has a 1-bp insertion and *Csclv3#2* has a 1-bp deletion, leading to premature termination of protein translation and a frameshift mutation, respectively (Fig. 1A and B). No mutations were detected in the potential off-target sites of the PCR sequencing products (Supplementary Data Table S2). Compared with wild type (WT), the *Csclv3* mutants displayed dramatically increased numbers of floral organ, including sepals, petals, stamens, and carpels (Fig. 1C–I). WT flowers generally produce 5 petals, 5 stamens, and 3 carpels, while *Csclv3* flowers exhibit 9–11 petals, 10–12 stamens, and 8–10 carpels. The floral phenotype is much severe than that of RNAi lines previously reported [31]. More interestingly, the fruits of *Csclv3* mutants were shorter and wider, displaying stumpy fruit with significantly reduced fruit shape index compared with WT (Fig. 1J–M).

### *CsCLV1* negatively regulates floral organ number but positively controls fruit shape index in cucumber

**Figure 2:**
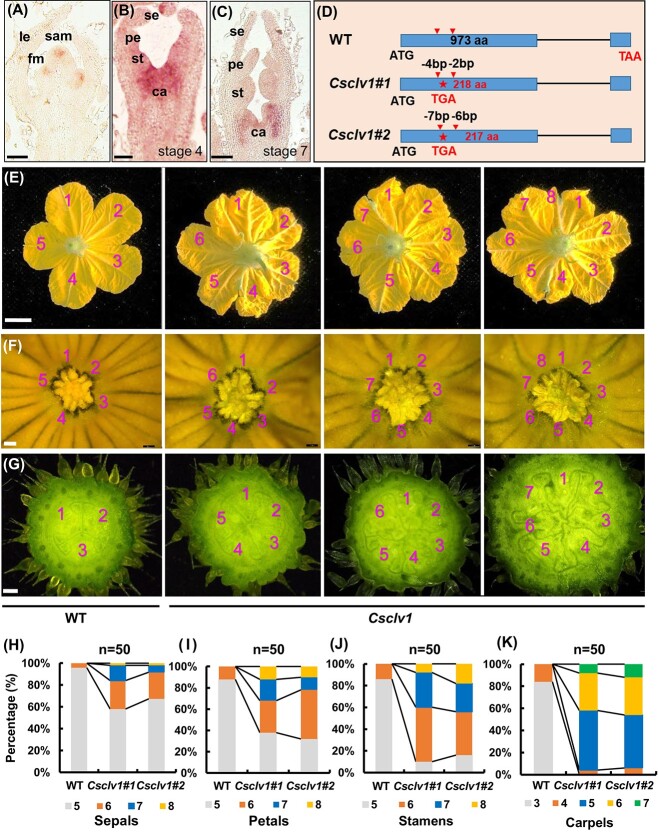
Expression analysis of *CsCLV1* and phenotype identification of floral organ numbers in *Csclv1* mutants in cucumber. **A**–**C**  *In situ* hybridization analysis of *CsCLV1* in SAM and FM of different stages. le, leaf or leaf primordium; fm, floral meristem; se, sepal primordium; pe, petal primordium; st, stamen primordium; ca, carpel primordium. Scale bars, 100 μm. **D** Two mutation sites in *CsCLV1* generated by CRISPR/Cas9, both of which result in premature termination of protein translation, with red arrows representing the two target sites and red stars indicating where the protein's translation is prematurely terminated. **E**–**G** Representative phenotypes of petals (**E**), stamens (**F**), and carpels (**G**) in *Csclv1#1* mutants and WT. Scale bars = 1 cm in (**E**) and 100 μm in (**F** and **G**). **H**–**K** Percentages of sepals (**H**), petals (**I**), stamens (**J**), and carpels (**K**) in WT and *Csclv1* lines (*n* = 50).

To investigate the downstream signaling components of CsCLV3 peptide, we chose its receptor *CLV1* homolog (Cs*CLV1*) in cucumber for characterization. qRT–PCR demonstrated that *CsCLV1* was expressed ubiquitously in different organs (Supplementary Data Fig. S1A). *In situ* hybridization showed that *CsCLV1* was enriched in the SAM and floral meristem (FM), especially in the deep layer of the central zone (Fig. 2A, Supplementary Data Fig. S1C), similar to *CLV1* in *Arabidopsis* [4]. *CsCLV1* signals were also detected in floral organ primordia, with strong signals in developing carpels, nectaries, and ovules (Fig. 2B and C, Supplementary Data Fig. S1C–H). No signals were detected in the negative control (Supplementary Data Fig. S1B).

To explore the biological function of *CsCLV1* in cucumber, we engineered mutations in *CsCLV1* with two single-guide RNAs (sgRNAs) using the CRISPR/Cas9 system. Two homozygous mutants were obtained*, Csclv1#1* (deletions of 4 and 2 bp) and *Csclv1#2* (deletions of 7 and 6 bp), both resulting in premature termination (Fig. 2D, Supplementary Data Fig. S1I). No mutations were detected in the potential off-target sites of the PCR sequencing products (Supplementary Data Table S3). Similarly to *Csclv3* mutants, *Csclv1* mutants showed significantly increased floral organ numbers. The average numbers of sepals, petals, stamens, and carpels were 5.0, 5.1, 5.1, and 3.2 in WT, while in the *Csclv1* mutants they were 5.6, 6.0, 6.4, and 5.5, respectively (Fig. 2E-K).

**Figure 3:**
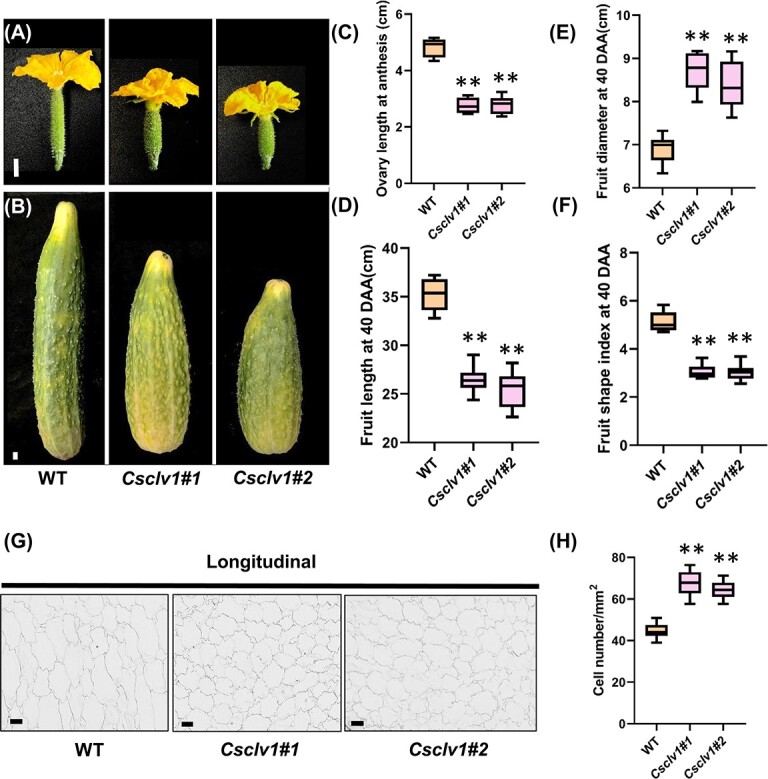
Functional disruption of *CsCLV1* led to stumpy fruit in cucumber*.*  **A**, **B** Cucumber ovaries at anthesis (**A**) and fruits at 40 DAA (**B**) in WT and *Csclv1* mutants, Scale bars, 1 cm. **C**, **D** Quantification of ovary length at anthesis (**C**) and fruit length at 40 DAA (**D**) in WT and *Csclv1* mutants. Values are means ± standard deviation (*n* = 8). **E**, **F** Quantification of fruit diameter (**E**) and fruit shape index (**F**) in WT and *Csclv1* mutants at 40 DAA. Values are means ± standard deviation (*n* = 8). **G** Representative images of longitudinal sections of cucumber fruit at 40 DAA in WT and *Csclv1* mutants (scale bars, 100 μm). **H** Number of cells per unit area in longitudinal sections of fruit at 40 DAA in WT and *Csclv1* mutants. Values are means ± standard deviation (*n* = 15). ^**^P < 0.01 (two-tailed Student’s *t*-test).

Notably, fruit length was significantly reduced in *Csclv1* mutants (Fig. 3A and B), with an average of 42.7% decrease at anthesis (Fig. 3C), and 26.8% reduction at 40 DAA compared with WT (Fig. 3D). However, the average fruit diameter of *Csclv1* mutants increased by 24.0% at 40 DAA (Fig. 3E). Consequently, the fruit shape index was significantly reduced in *Csclv1* mutants (Fig. 3F). To investigate the reason for reduced fruit length in *Csclv1* mutants, longitudinal sections of fruits at 40 DAA were analyzed. Our data indicated that cell size was greatly decreased in the *Csclv1* mutants compared with WT (Fig. 3G and H), implying that *CsCLV1* promotes fruit elongation through cell expansion in cucumber.

### CsCLV1 physically interacts with CsGPA1 at protein level

**Figure 4:**
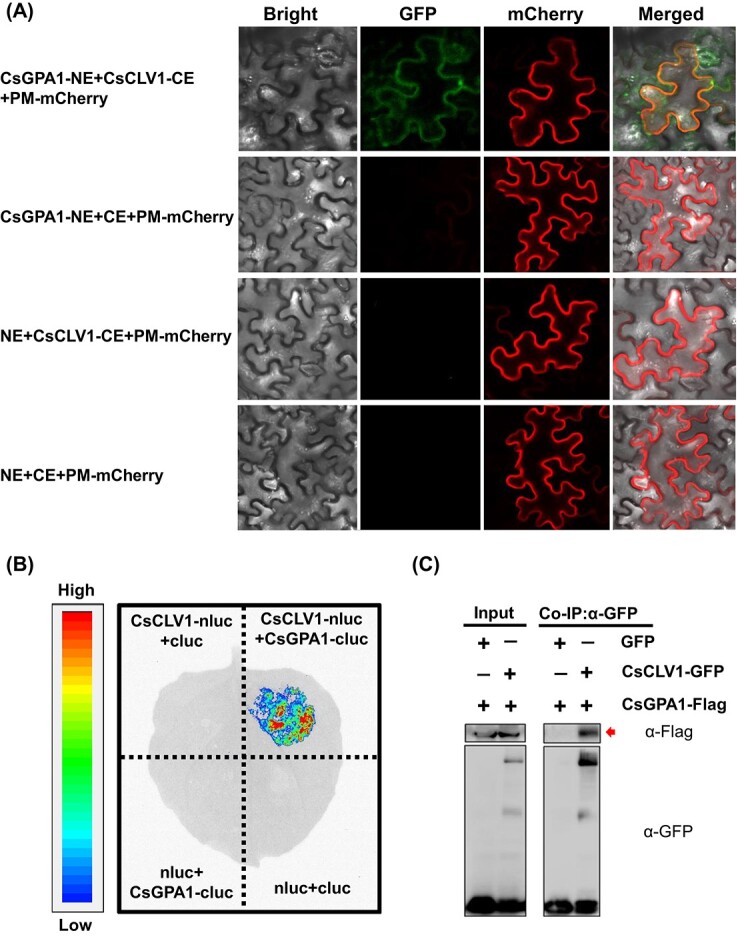
CsCLV1 physically interacts with CsGPA1 at protein level. **A** BiFC assay. CsGPA1-YFP^N^, CsCLV1-YFP^C^, and PM-mCherry were co-infiltrated into *N. benthamiana* leaves. The other combination was used as a control. Green fluorescence represents the interaction signal, red fluorescence represents the plasma membrane marker signal, and orange fluorescence represents the merged outcome. **B** Firefly LCI assay. CsCLV1-nluc and CsGPA1-cluc were co-infiltrated into *N. benthamiana* leaves and the remainder of the combinations were used as controls. Imaging in red represents strong interactions and blue indicates weak interactions. **C** Co-IP assay. The constructs specified were expressed in leaves of *N. benthamiana*, and Co-IP was performed using anti-GFP antibody. The band indicated by the red arrow represents the *in vivo* interaction between CsCLV1 and CsGPA1.

To further identify the signaling components of the CsCLV3–CsCLV1 pathway, we screened for CsCLV1 companions using a yeast two-hybrid library based on its intracellular kinase domain (Supplementary Data Fig. S2A). Sequencing and annotation of 116 positive clones were captured (Supplementary Data Table S1). Among them, the guanine nucleotide-binding protein α-subunit (GPA1) and GTP-binding nuclear protein Ran were identified (Supplementary Data Table S1). The *Gα-subunit* (*CT2*) had been previously documented as a participant in the CLV signaling pathway by affecting meristem size in maize [27]. We therefore speculated whether the Gα-subunit (designated CsGPA1 hereinafter) transmits CLV signaling in cucumber. The interaction between the kinase domain of CsCLV1 and CsGPA1 was further verified by yeast two-hybrid assay (Supplementary Data Fig. S2B). The bimolecular fluorescence complementation (BiFC) assay indicated that CsCLV1 and CsGPA1 interact at the plasma membrane. Subsequently, firefly luciferase complementation imaging (LCI) and co-immunoprecipitation (Co-IP) assays further confirmed the interaction of CsCLV1 and CsGPA1 (Fig. 4A–C). Therefore, CsGPA1 could physically interact with CsCLV1 at protein level.

### Functional disruption of *CsGPA1* produced a similar phenotype to *Csclv1* mutants

**Figure 5:**
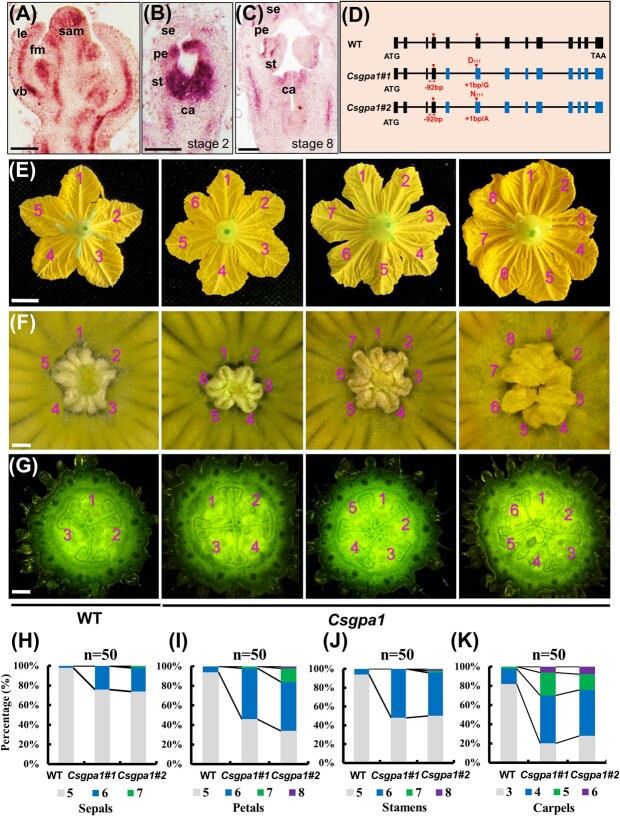
Expression analysis of *CsGPA1* and phenotypic analysis of floral organ numbers in *Csgpa1* mutants. **A**–**C**  *In situ* hybridization analysis of *CsGPA1* in SAM and FM at different stages. le, leaf or leaf primordium; fm, floral meristem; se, sepal primordium; pe, petal primordium; st, stamen primordium; ca, carpel primordium; vb, vascular bundle. Scale bars, 250 μm. **D** Mutant alleles generated in *Csgpa1* mutants by CRISPR/Cas9 in cucumber. The red inverted triangles represent the two targets located in the fourth and sixth exons of *CsGPA1*. The fourth exon resulted in a 92-bp deletion including the intron in both mutants, leading to loss of the fourth exon. A base was added in the sixth exon (+G and +A, respectively) and resulted in translation into different amino acids, D (aspartic acid) and N (asparagine) at the 111th position, respectively. Both forms led to frameshift mutation. Blue bars represent amino acids translated in *Csgpa1* mutants, different from WT. **E**–**G** Representative phenotypes of petals (**E**) and stamens (**F**) and carpel numbers (**G**) in WT and *Csgpa1#2* mutants. Scale bars = 1 cm in (**E**) and 100 μm in (**F** and **G**). **H**–**K** Percentages of sepals (**H**), petals (**I**), stamens (**J**), and carpels (**K**) in WT and *Csgpa1* lines (*n* = 50).

Phylogenetic analysis showed that GPA1 homologs in dicotyledons were clustered into a clade distinct from than those in monocotyledons (Supplementary Data Fig. S2C). *CsGPA1* was expressed ubiquitously in various tissues of cucumber (Supplementary Data Fig. S2D). *In situ* hybridization demonstrated that *CsGPA1* signals were strongly enriched in the SAM, FM, vascular bundles of leaf primordia and floral organs (Fig. 5A–C, Supplementary Data Fig. S2E–H). Transcripts of *CsGPA1* were also detected in developing nectaries and ovules (Supplementary Data Fig. S2I). No signals were detected in the negative control (Supplementary Data Fig. S2E). Notably, the expression pattern of *CsGPA1* largely overlapped with that of *CsCLV1*, implying that they may function in the same pathway in cucumber.

**Figure 6:**
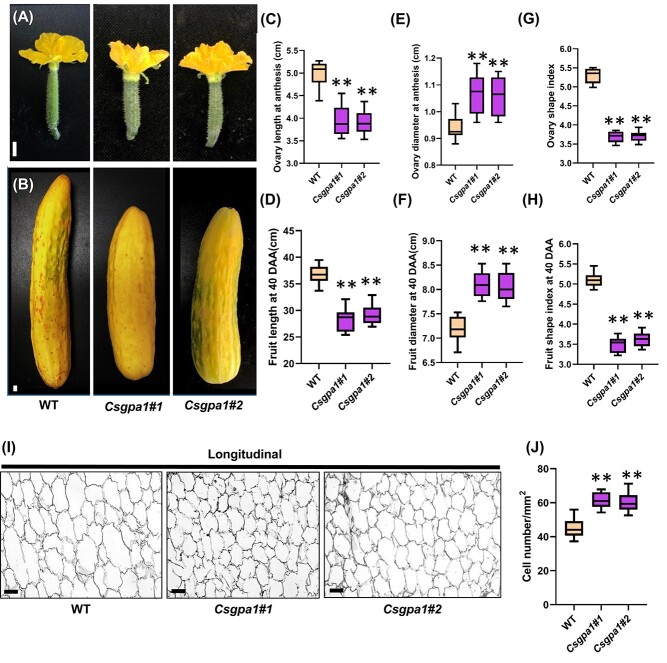
Phenotypic analysis of fruits in *Csgpa1* mutants. **A**, **B** Cucumber ovaries at anthesis (**A**) and fruits at 40 DAA (**B**) in WT and *Csgpa1* mutants, Scale bars, 1 cm. **C**, **D** Quantification of cucumber ovary length at anthesis (**C**) and fruit length at 40 DAA (**D**) in WT and *Csgpa1* mutants. Values are means ± standard deviation (*n* = 8). **E**–**H** Fruit diameter (**E**, **F**) and fruit shape index (**G**, **H**) in WT and *Csgpa1* mutants at anthesis (**E**, **G**) and 40 DAA (**F**, **H**). Values are means ± standard deviation (*n* = 8). **I** Representative images of longitudinal sections of cucumber fruit at 40 DAA in WT and *Csgpa1* mutants (scale bars, 100 μm). **J** Number of cells per unit area in longitudinal sections of fruit at 40 DAA in WT and *Csgpa1* mutants. Values are means ± standard deviation (*n* = 15). ^**^P < 0.01 (two-tailed Student’s *t*-test).

Next, we explored the biological functions of *CsGPA1* via the CRISPR/Cas9 system. Two homozygous mutant lines, *Csgpa1#1* and *Csgpa1#2*, were obtained, both leading to frameshift mutations (Fig. 5D, Supplementary Data Fig. S2J). No mutations were detected in the potential off-target sites of the PCR sequencing products (Supplementary Data Table S4). Phenotypic analyses revealed a significantly elevated number of floral organs in *Csgpa1* mutants compared with WT. In WT, the average numbers of sepals, petals, stamens, and carpels were 5.0, 5.1, 5.1 and 3.2, while they were 5.3, 5.7, 5.5, and 4.1 in *Csgpa1* mutants, respectively (Fig. 5E–K). Furthermore, both *Csgpa1* mutants produced significantly shorter and wider fruits compared with WT (Fig. 6A and B). Fruit length decreased by 21.0% at anthesis and 21.7% at 40 DAA in *Csgpa1* mutants (Fig. 6C and D). However, fruit diameter increased by 12.5% at anthesis and 13.2% at 40 DAA compared with WT (Fig. 6E and F), and the fruit shape index was significantly decreased in both stages of *Csgpa1* mutants (Fig. 6G and H). Fruit longitudinal sections indicated that cell size was greatly reduced in *Csgpa1* mutants (Fig. 6I and J). These phenotypes were similar to that of *Csclv1*, supporting the idea that CsGPA1 may function in the same pathway of CsCLV1 during flower and fruit development in cucumber.

In addition, the hypocotyls of *Csgpa1* mutants were significantly shorter than those of WT (Supplementary Data Fig. S3A and B). Hormonal measurements demonstrated that auxin and gibberellin levels were greatly decreased in *Csgpa1* mutant hypocotyls (Supplementary Data Fig. S3C and D). Similar to the phenotype of *gpa1* (Gα-subunit) and *agb1* (Gβ-subunit) mutants in *Arabidopsis*. We also observed the round leaf shape phenotype in *Csgpa1* mutants compared with the palmate leaf in WT (Supplementary Data Fig. S3E) [32].

### Expression of *CsWUS* was elevated in the meristem of *Csgpa1* mutants

**Figure 7:**
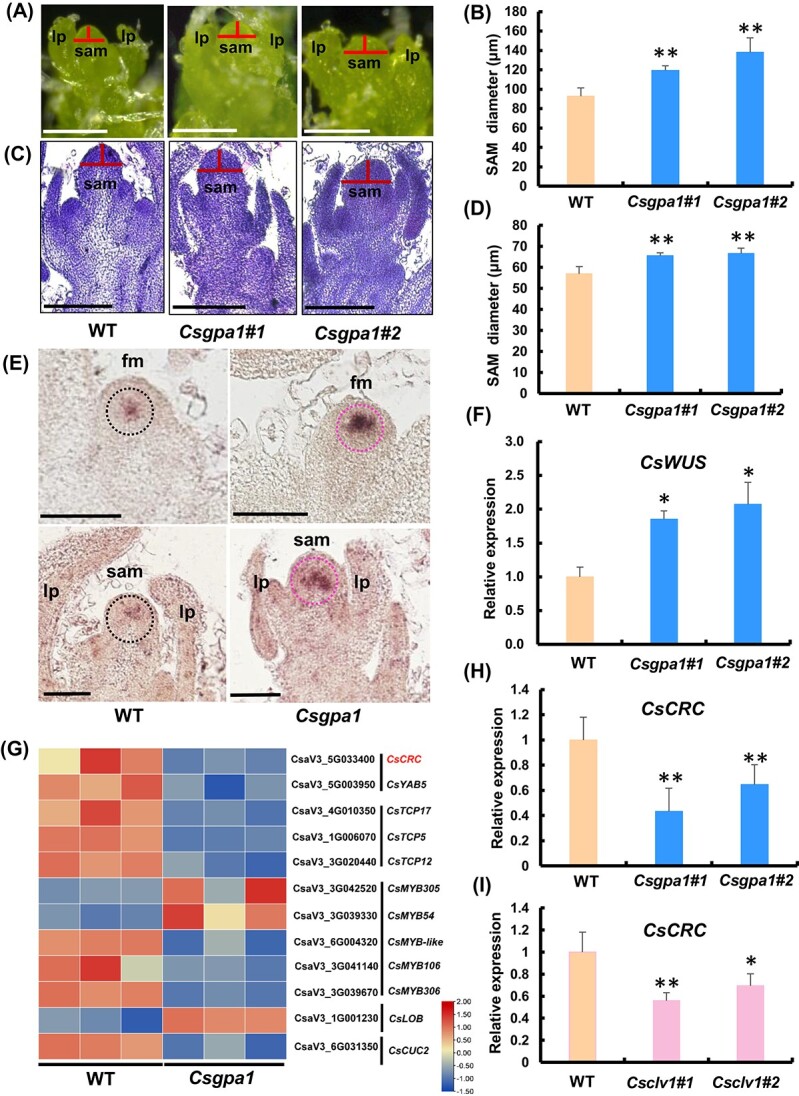
*CsGPA1* affects the expression of *CsWUS* and *CsCRC* in cucumber. **A** Stereoscopic images of SAM of 20-day-old cucumber seedlings from WT and *Csgpa1* mutants (scale bars, 0.25 mm). lp, leaf primordium. **B** SAM diameter in stereoscopic images (scale bars, 100 μm). **C** Longitudinal sections of shoot apexes from 20-day-old WT and *Csgpa1* cucumber seedlings. **D** SAM diameter in longitudinal sections from shoot apexes of WT and *Csgpa1* mutants. **E**, **F**  *CsWUS* expression was detected in the shoot apex of 20-day-old cucumber seedlings from WT and *Csgpa1* mutants by *in situ* hybridization (**E**) and qRT–PCR (**F**) (scale bars, 100 μm). **G** Heat map of the related DEGs of transcription factors in WT and *Csgpa1* lines. Members of different gene families are separated by vertical lines. **H**, **I** Gene expression analysis of *CsCRC* in *Csgpa1* mutants (**H**) and *Csclv1* mutants (**I**). ^*^P < 0.05, ^**^P < 0.01 (two-tailed Student’s *t*-test).

Given that the maize *GPA1*-homologous gene *CT2* regulates shoot meristem size by participating in the CLV signaling pathway [27, 33], we explored shoot meristem development in *Csgpa1* mutants using light microscopy and sectioning. Our data showed that shoot meristem size was significantly enlarged (an average of 36.3% increase) in diameter in *Csgpa1* mutants compared with WT (Fig. 7A–D). Considering that *CsWUS* was reported to be the key determinant of SAM size [34], the expression of *CsWUS* was detected by *in situ* hybridization and qRT–PCR (Fig. 7E and F). As expected, the *CsWUS* signal was greatly enhanced in the FM and SAM of *Csgpa1* compared with WT (Fig. 7E). qRT–PCR data indicated that the expression level of *CsWUS* increased 2.0-fold in the *Csgpa1* shoot apex (Fig. 7F). Therefore, the increment of floral organ numbers in *Csgpa1* may be due to elevated *CsWUS* expression and enlarged SAM size in cucumber.

### Expression of *CsCRC* was decreased in *Csgpa1* and *Csclv1* mutant fruits

To explore the underlying factors of shorter fruit phenotype in the *Csgpa1* mutant, RNA-seq analysis was conducted on ovaries at anthesis from *Csgpa1* mutants and WT. The results revealed that 61 and 143 differentially expressed genes (DEGs) were up- and downregulated in *Csgpa1* mutants versus WT (fold change ≥ 2, FDR < 0.01), respectively (Supplementary Data Table S2, Supplementary Data Fig. S4A). Among the 204 DEGs, transcription factors were enriched, including YABBY, MYB, and zinc finger family genes (Supplementary Data Fig. S4B and C). Notably, the YABBY transcription factor *CRABS CLAW* (*CsCRC*) was significantly downregulated in *Csgpa1* mutant fruits (Fig. 7G). Our previous study showed that *CsCRC* is a major-effect fruit length regulator that stimulates fruit elongation by cell expansion in cucumber [35]. qRT–PCR demonstrated that the expression level of *CsCRC* declined 1.9-fold in *Csgpa1* and 1.6-fold in *Csclv1* (Fig. 7H and I). Therefore, CsCLV1 and CsGPA1 might participate in fruit elongation by modulating *CsCRC* transcription in cucumber.

## Discussion

### The Gα-subunit (*CsGPA1*) is a novel regulator of floral organ number and fruit shape in cucumber

Heterotrimeric G proteins are important signaling molecules that help transfer signals from outside to inside the cell by interacting with G-protein-coupled receptors (GPCRs) [24]. However, due to lack of GPCRs in plants, G proteins interact with single transmembrane receptors to regulate plant growth and development [36]. The functions of G proteins have been intensively explored in stress responses [25, 37], but there are relatively few reports in plant organ development [36]. In dicots, the function of *GPA1* in organ development has only been investigated in *Arabidopsis*, in which mutants of *gpa1* displayed stumpy hypocotyls, smaller flowers, and slightly shortened siliques, but no changes in SAM size and floral organ numbers [22, 23, 32]. In our study, we found that *CsGPA1* is expressed in the SAM, FM, vascular bundles of leaf primordia, and floral organs (Fig. 5A–C, Supplementary Data Fig. S2E–H), similar to *GPA1* in *Arabidopsis* [38]. In cucumber *gpa1* mutants, we also found the round leaf shape and short hypocotyl phenotypes as found in *Arabidopsis* (Supplementary Data Fig. S3A, B, and E) [23]. Interestingly, we showed that CsGPA1 could interact with receptor kinase CsCLV1 at protein level (Fig. 4, Supplementary Data Fig. S2B). Functional disruption of *CsCLV1* resulted in flowers with extra organs and stumpy fruits (Figs 2E–K and 3A–F). Loss of function of *CsGPA1* led to phenotypes similar to *Csclv1* mutant (Figs 5E–K and 6A–H), suggesting that CsGPA1 is a novel player in floral organ number and fruit shape development in the CLV signaling pathway in cucumber.

### *CsGPA1* negatively regulates floral organ numbers by modulating shoot apical meristem size

**Figure 8:**
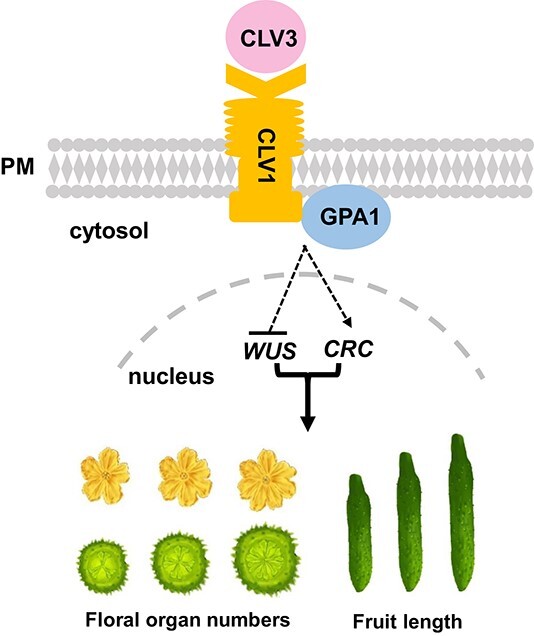
A model for the regulation of floral organ number and fruit length by CsGPA1 in the CLV signaling pathway. The CsCLV3 peptide is received by receptor kinase CsCLV1. Gα-subunit CsGPA1 protein interacts with CsCLV1 to inhibit floral organ number but promote fruit elongation, by repressing *CsWUS* expression and activating *CsCRC* transcription in cucumber.

Variations in floral organ numbers are usually due to two reasons, one being change in the size of the meristem, and the other being homeotic conversion of floral organs [39]. Genes in the CLV–WUS signaling pathway were shown to regulate floral organ numbers by affecting meristem size [34]. Elevated *WUS* expression led to an enlarged meristem and extra floral organs in tomato and maize, and overexpression of *CsWUS* resulted in more carpels in cucumber [16, 17, 31]. Here, we found that CsGPA1 interacts with the CsCLV1 receptor kinase in cucumber. Functional disruption of *CsGPA1* resulted in extra floral organs, increased meristem size, and enhanced *CsWUS* expression (Figs 5E–K and 7A–F), suggesting that CsGPA1 negatively regulates floral organ numbers probably by inhibiting *CsWUS*-mediated meristem size in cucumber (Fig. 8).

### The CsCLV1–CsGPA1 module positively regulates cucumber fruit elongation

The *CRC* gene belongs to the YABBY family of transcription factors containing the C_2_C_2_ zinc-finger and DNA-binding domain, which has been reported to be involved in carpel and nectary development in *Arabidopsis* [40–43]. In our previous study, *CsCRC* was found to promote fruit elongation through cell expansion [35, 44]. Here, we found that fruit length was significantly decreased in *Csclv1* and *Csgpa1* mutants (Figs 3A–D and 6A–D). Sectioning results showed that the reduced fruit length was caused by smaller cell (Figs 3G and H and  6I and J). Notably, *CsCRC* expression was significantly reduced in both *Csclv1* and *Csgpa1* mutants (Supplementary Data Table S2, Fig. 7H and I). The expression patterns of *CsCRC* overlap with those of *CsGPA1* and *CsCLV1*, with particularly enriched signal in developing carpel primordia and nectaries (Figs 2B and C and  5B and C, Supplementary Data Figs S1D–J and S2G-I [35]. These data suggest that CsCLV1 and CsGPA1 act as a module to stimulate fruit elongation, probably through *CsCRC*-mediated cell expansion in cucumber (Fig. 8).

In conclusion, we identified a new player, CsGPA1, in the CLV signaling pathway during flower and fruit development in cucumber. Specifically, the CsCLV3 peptide was received by receptor kinase CsCLV1. Gα-subunit CsGPA1 protein interacts with CsCLV1 to inhibit floral organ number but promote fruit elongation, by repressing *CsWUS* expression and activating *CsCRC* transcription (Fig. 8). Our findings not only enrich the gene regulatory network downstream of the CLV signaling pathway, but also provide target genes for precise manipulation of fruit shape during cucumber breeding.

## Materials and methods

### Plant materials

Cucumber (*Cucumis sativus* L.) inbred line 9930 of the North China type was used in this study. Soaked cucumber seeds were transferred to plugs after 36 h of germination in an incubator at 28°C under dark conditions and then transplanted to the greenhouse of China Agricultural University in Beijing when the third true leaf appeared. Standard water and fertilizer management as well as pest control were applied for all cucumber seedlings. *Nicotiana benthamiana* plants were grown in a light incubator with 16 h light/8 h dark at 25°C. Tobacco seedlings at 6 weeks old grown under standard management were used for biochemical analysis.

### RNA extraction and quantitative real-time PCR

Different tissue samples were collected and quickly placed in liquid nitrogen for extracting total RNA using the Eastep^®^ Super Total RNA Extraction Kit (Promega). Two micrograms of RNA was used for synthesizing cDNA with the FastKing gDNA Dispelling RT SuperMix Kit (Tiangen, Beijing, China). The qRT–PCR assay was performed using TB Green^®^ Premix Ex TaqTM II (Takara, Kyoto, Japan) on the CFX384 Real-Time PCR Detection System (Bio-Rad) for gene expression analysis. Each gene had three biological replicates and three technical replicates. *CsUBI* was used as an internal reference gene [45]. Detailed information on the primers is listed in Supplementary Data Table S6.

### *In situ* hybridization

The shoot apexes of 20- and 35-day-old cucumber seedlings and young ovaries were harvested. Samples were fixed, sectioned, and hybridized for detecting gene expression. The detailed process was performed as previously reported [46]. Specific regions of target genes were used to design probe primers, after which sense and antisense probes were synthesized by SP6 and T7 RNA polymerase using the DIG RNA Labeling Kit (Roche, Basel, Switzerland), respectively. Different flower developmental stages were classified with reference to a previous study [47]. The probe primer sequence is listed in Supplementary Data Table S6.

### Cucumber genetic transformation

For the construction of knockout vectors, sgRNA-specific sequences of target genes were selected firstly on the web (http://crispr.hzau.edu.cn/CRISPR2/). PCR fragments containing two targets were generated by two pairs of primers amplified using pCBC-DT1T2 vector as template, and then inserted into the binary CRISPR/Cas9 vector pKSE402 with the BsaI site and T4 ligase. After the recombinant pKSE402 vector was chemically transferred to *Agrobacterium tumefaciens* strain EHA105, transgenic cucumber plants were obtained by *Agrobacterium*-mediated cucumber cotyledon transformation as previous described [48]. *T*_0_ transgenic plants were selected by screening cucumber seedlings containing GFP fluorescence using a fluorescence microscope, and the transgenic mutant form was verified by sequencing the PCR products of target genes. The primer information is listed in Supplementary Data Table S6.

### Yeast two-hybrid assay

The intracellular kinase domain of *CsCLV1* was integrated into the pGBKT7 vector, then employed as a bait protein for hybridization with a cucumber yeast library of pGADT7, following a self-activation assay. Positive clones were identified by PCR amplification and sequencing after screening on selective medium SD/−Trp/−Leu/-His/−Ade with X-α-Gal. In yeast two-hybrid assays, the coding sequence of *CsGPA1* was introduced into the pGADT7 vector. AD and BK recombinant vectors were co-transfected into the yeast strain AH109 and grown on SD/−Trp/−Leu medium. Protein interaction was screened on selective medium SD/−Trp/−Leu/−His/−Ade. The specific procedure was performed as described in the Matchmaker™ GAL4 Two-Hybrid System 3 & Libraries (Clontech) protocol. The primer information can be found in Supplementary Data Table S6.

### Bimolecular fluorescence complementation assay

The full-length coding sequences without the stop codon of *CsCLV1* and *CsGPA1* were introduced into *pSPYCE-35S* and *pSPYNE-35S* vectors, respectively. Recombinant vectors were transferred into *Agrobacterium* strain GV3101 and co-injected into 6-week-old *N. benthamiana* leaves together with the p19 *Agrobacterium* and plasma membrane PM-mCherry as described [49]. The YFP signals were detected by confocal microscopy (Olympus BX51, Japan) under an excitation wavelength of 488 nm for interaction signals and 562 nm for PM-mCherry at 48 h after infiltration. The primers used are shown in Supplementary Data Table S6.

### Luciferase complementation imaging assay

The complete coding sequence without the stop codon of *CsCLV1* was inserted into pCAMBIA1300-nLUC and the full-length coding sequence of *CsGPA1* was integrated into pCAMBIA1300-cLUC. The recombinant vectors were then introduced into *Agrobacterium* strain GV3101 and co-injected with p19 *Agrobacterium* into 6-week-old *N. benthamiana* leaves. After 48 h of infiltration, the abaxial surfaces of the leaves were treated with a 1 mM solution of d-Luciferin Potassium Salt (Biovision). After a period of 5 min in darkness, the interactions were captured using a CCD imaging system (MiniChemi 610, Sagecreation). Details of the primer sequences have been provided in Supplementary Data Table S6.

### Co-immunoprecipitation assay

The entire coding sequence of *CsCLV1*, excluding the stop codon, was inserted into pCAMBIA1300-GFP vector and the full-length coding sequence without the stop codon of *CsGPA1* was cloned into pCAMBIA1300-FLAG vector. The recombinant vectors were then transferred into *Agrobacterium* strain GV3101 and co-infiltrated with p19 *Agrobacterium* into *N. benthamiana* leaves. Samples were collected and ground into powder using liquid nitrogen after a 48-h infiltration. Following this, the samples were homogenized in extraction buffer. Immunoprecipitation was carried out using anti-GFP antibody-coated agarose beads (KTHEALTH, China; KTSM1301) at 4°C for 3 h. The beads underwent six washes with washing buffer (50 mM HEPES [pH 7.5], 150 mM KCl, 1 mM EDTA, 0.2% [v/v] Triton-X 100, 1 mM DTT). The immunoprecipitates were separated using SDS–PAGE and detected by immunoblot with either anti-GFP (TransGen Biotech, Beijing, China; Catalog No. HT801) or anti-FLAG (Sigma–Aldrich, Burlington, MA, USA; Catalogue No. F3165) antibodies. The primer information can be found in Supplementary Data Table S6.

### Histology

Fruit tissue samples were prepared at 40 days after anthesis (DAA) by fixation, embedding, and sectioning to 10-μm thickness, followed by dewaxing. Subsequently, images were captured using an Olympus light microscope. Cell counting was performed in various fields, and the number of cells per unit area was calculated. For each sample, three fields were observed, and five biological samples were examined for every representative line.

### Measurement of endogenous hormones

To analyze the amounts of auxin (IAA) and gibberellic acid (GA3), we harvested and homogenized 0.1–0.3 g of hypocotyls from 10-day-old seedling samples of WT and *Csgpa1* mutants. This was carried out in 4 ml of 80% methanol containing an antioxidant. Enzyme-linked immunosorbent assays (ELISAs) were performed to extract and quantify phytohormones, as previously described [50]. Five biological replicates were measured for each line.

### Transcriptome analysis

Ovaries at anthesis from WT and *Csgpa1* were utilized for RNA-seq, with each sample consisting of three biological replicates. The Biomarker Technologies Corporation (Beijing, China) performed RNA library construction and sequencing with an Illumina NovaSeq 6000 platform. Transcriptomic data were analyzed on the BMKCloud platform (www.biocloud.net) according to previously stated methods [51]. DEGs were identified using DESeq2 with parameter (fold change ≥ 2, FDR < 0.01). Supplementary Data Table S2 contains the information on sequencing data.

## Acknowledgements

This work was supported by grants from the National Key Research and Development Program of China (2022YFD1200502), the National Natural Science Foundation of China (32025033 and 31930097), Hunan High-level Talents Gathering Project-innovative Talents (2021RC5006), Pinduoduo-China Agricultural University Research Fund (PC2023B01002), and the 111 Project (B17043).

## Author contributions

X.Z., L.H., and Z.Z. designed the project. L.H. performed the experiments; L.H. and X.Z. wrote the paper; Y.H., C.L., D.T., and D.S. provided assistance in cucumber cultivation management and phenotype analysis; M.L., Z.W., J.C., L.L., S.W., W.S., L.W., C.G., T.W., and J.Z. provided experimental assistance; all the authors revised the manuscript.

## Data availability

The datasets have been submitted to the NCBI-SRA database under the BioProject PRJNA1088621.

## Conflict of interest

The authors declare no conflict of interest.

## Supplementary data

Supplementary data are available at *Horticulture Research* online.

## Supplementary Material

Web_Material_uhae110
