# Supplementary material for: Heterotrimeric Gα-subunit regulates flower and fruit development in CLAVATA signaling pathway in cucumber
Source: Hortic Res. 2024 Apr 16;11(6):uhae110. doi: 10.1093/hr/uhae110 (PMC11186068; doi:10.1093/hr/uhae110)
Supplement: Web_Material_uhae110 [file web_material_uhae110.zip › CsGPA1-Supplementary materials-revised.docx]

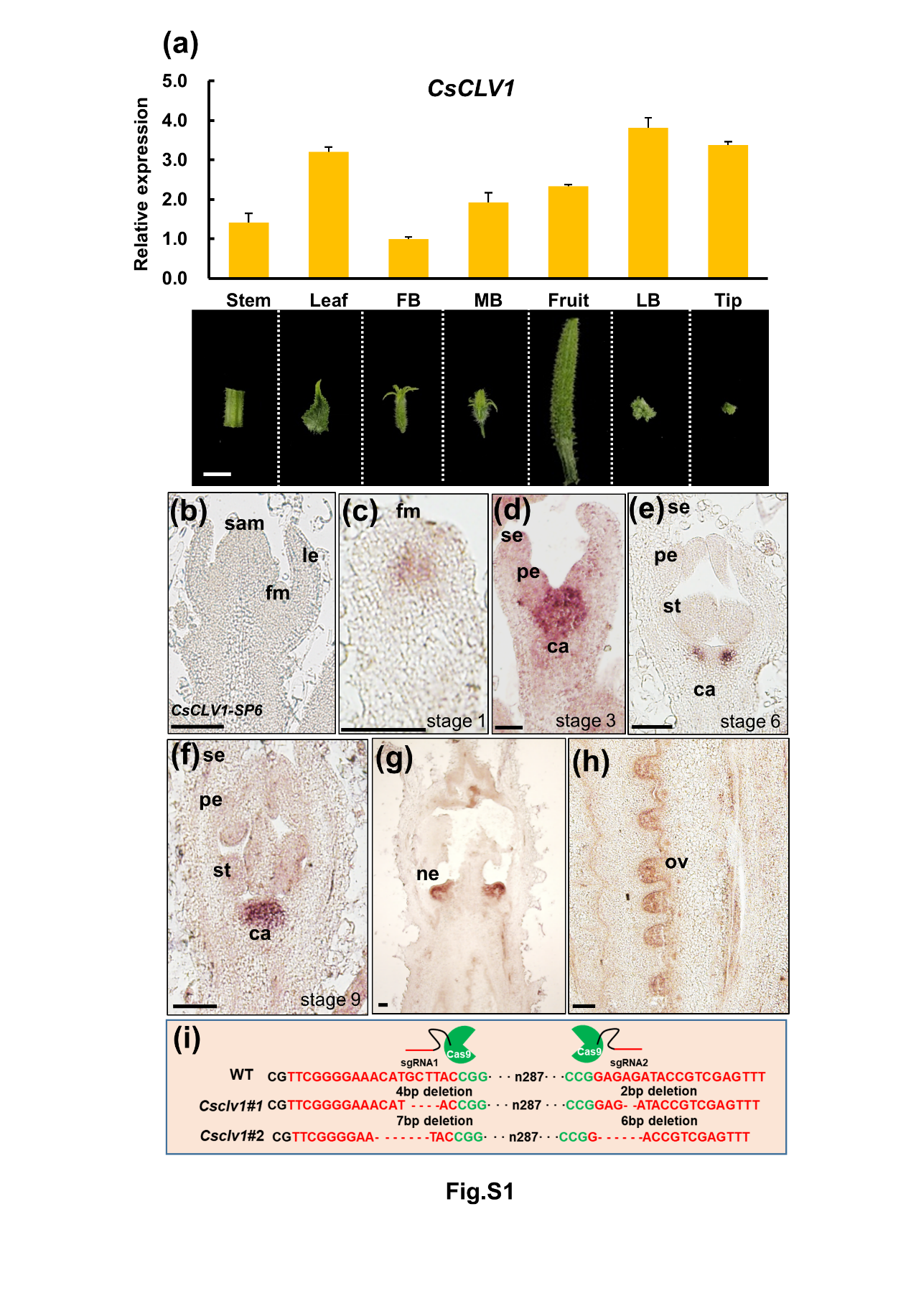


**Figure S1.** Expression pattern analysis of *CsCLV1* in cucumber.

(A) Evaluated expression of *CsGPA1* in different cucumber organs by qRT-PCR. (FB: Female bud/young ovary, MB: male bud, Fruit: ovary at anthesis, LB: lateral bud.) Scale bars, 1 cm. (B-H) *In situ* hybridization analysis of *CsCLV1* in SAM and FM of different stage. Note: le, leave or leaf primordia; fm, floral meristem; se, sepal primordium; pe, petal primordium; st, stamen primordium; ca, carpel primordium; ne, nectary; ov, ovule. Scale bars, 100 μm. (I) Mutation sites in *CsCLV1* generated via CRISPR/Cas9.


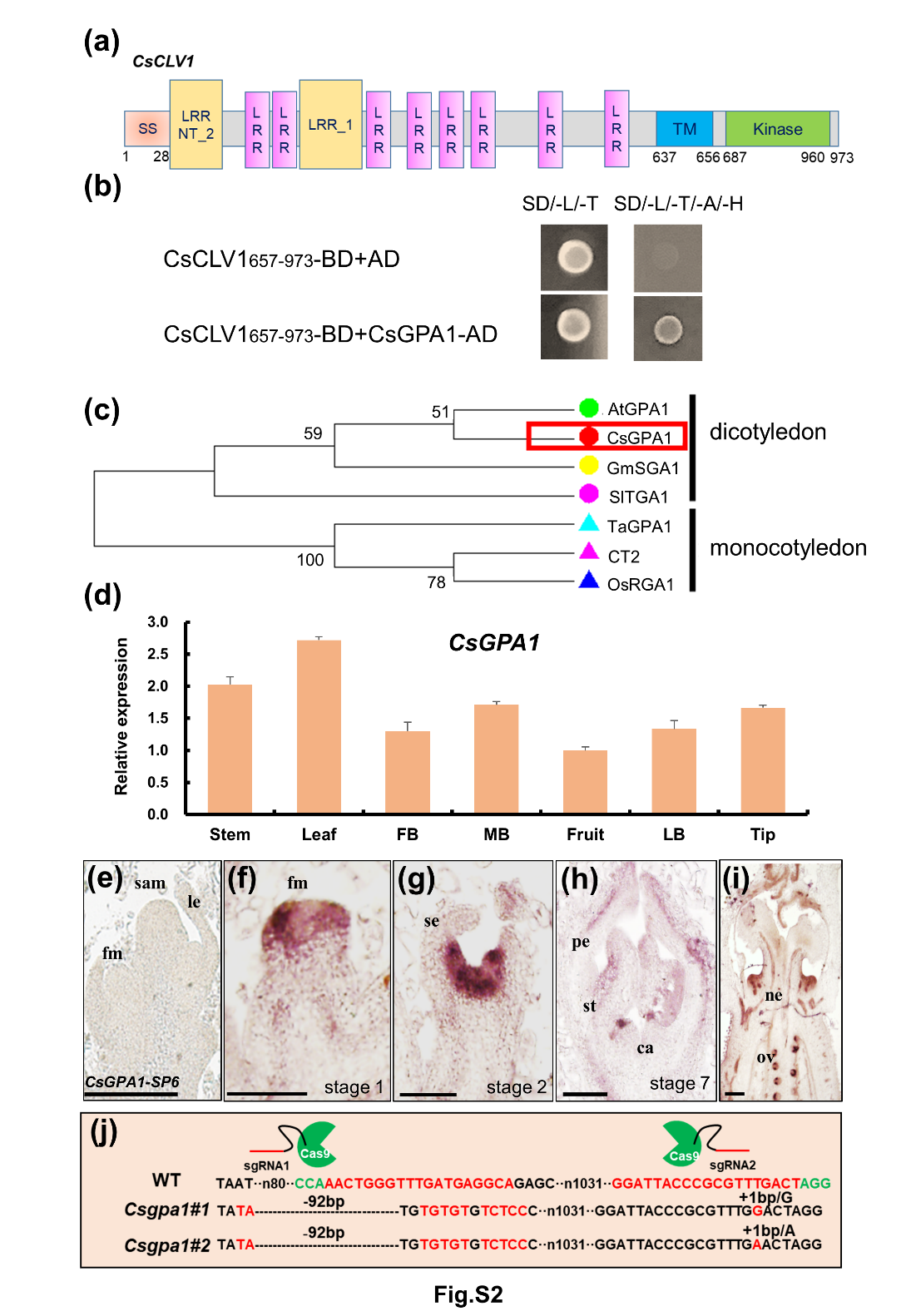


**Figure S2.** Identification and expression analysis of *CsGPA1* in cucumber.

(A) The gene structure of *CsCLV1*. (B) The intracellular kinase domain of CsCLV1 interacts with CsGPA1 by Yeast Two-Hybrid assay. (C) Phylogenetic analysis of homologous of CsGPA1 in monocotyledon (rice, maize, wheat) and dicotyledon (cucumber, tomato, Arabidopsis, soybean). The phylogenetic tree was plotted by the neighbor-joining method using MEGA5.0 software and repeated 1000 times. Numbers on branches indicate bootstrap values above 50. (D-I) Evaluation of *CsGPA1* expression by qRT-PCR (D) and *in situ* hybridization (E-I). Note: le, leave or leaf primordia; fm, floral meristem; se, sepal primordium; pe, petal primordium; st, stamen primordium; ca, carpel primordium; ne, nectary; ov, ovule. Scale bars, 250 μm. (J) Mutation sites in *CsGPA1* generated by CRISPR/Cas9.


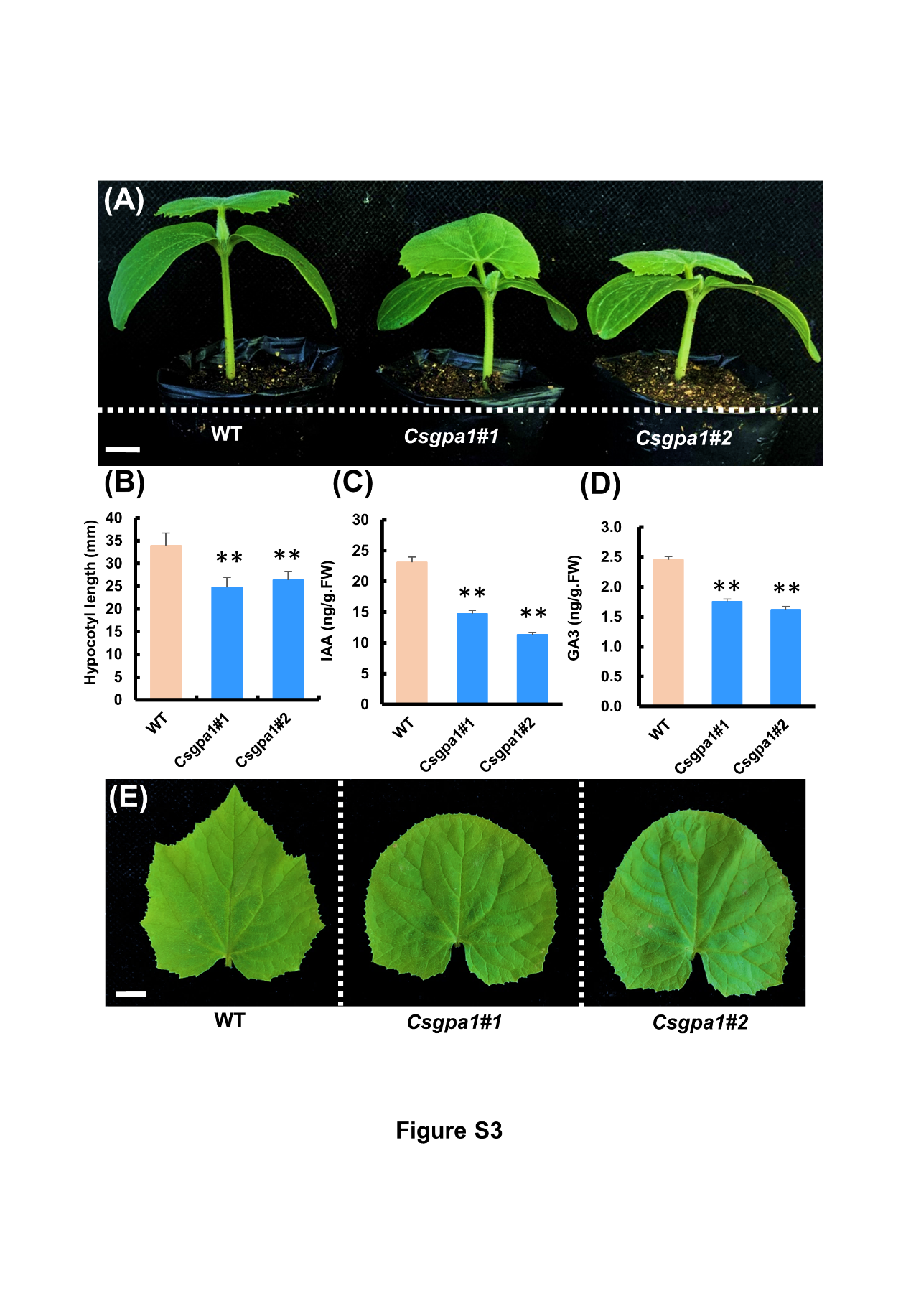


**Figure S3.** Phenotypic analysis of hypocotyl and leaf shape in *Csgpa1* mutants.

(A) Representative images of cucumber hypocotyl from WT and *Csgpa1* mutants (Scale bars, 1cm). (B) Quantification of hypocotyl length in WT and *Csgpa1* mutants of 15-day-old cucumber seedlings. (C-D) Measurement of endogenous hormones auxin (IAA) (C) and gibberellic acid (GA3) (D) from 10-day-old seedlings of WT and *Csgpa1* mutants. (E) Representative images of cucumber leaf shape from WT and *Csgpa1* mutants (Scale bars, 1 cm). Significance analysis of data differences was performed using the two-tailed Student’s *t*-test (* *p* < 0.05 and ** *p* < 0.01).


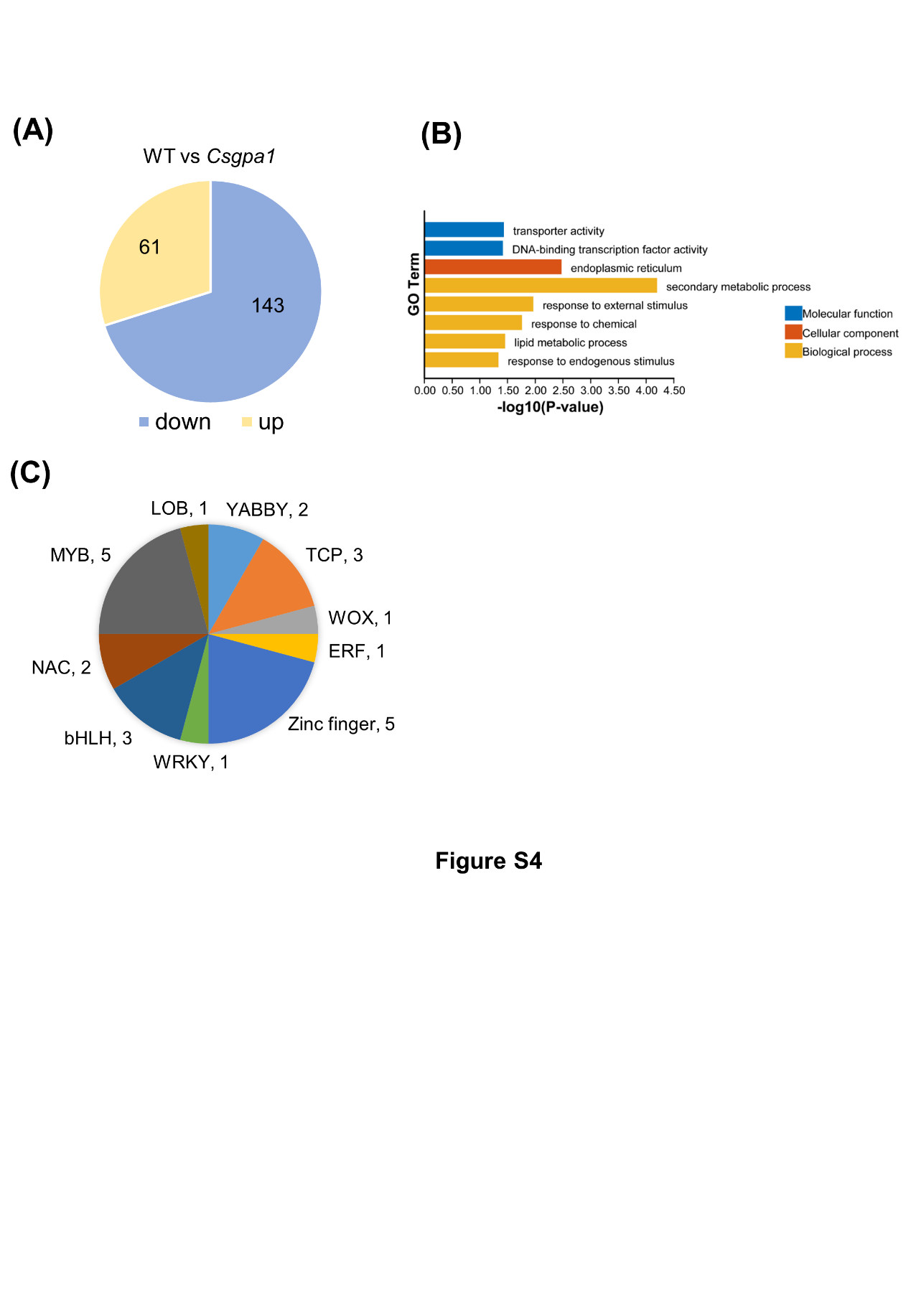


**Figure S4.** Transcriptome analysis in cucumber ovaries at anthesis of *Csgpa1* and WT.

(A) Distribution of DEGs between WT and *Csgpa1* mutant ovaries at anthesis. (B) Gene Ontology (GO) enrichment analysis of the DEGs in *Csgpa1*. (C) Functional category of the DEGs in *Csgpa1* encoding transcription factors. Significance analysis of data differences was performed using the two-tailed Student’s *t*-test (* *p* < 0.05 and ** *p* < 0.01).

**Table S2. Analysis of the potential off-target sites for *CsCLV3* sgRNA.**

| Targets | Potential off-target sites | | | | Presenceof mutation |
| --- | --- | --- | --- | --- | --- |
|  | **Name** | **Sequence** | **Gene** | **Number of mismatches** |  |
| Target1 | T1-off1 | AGAGGGGAGAGGTGGGGGG**CGG** | Csa3G199040 | 3 | none |
|  | T1-off2 | TGGGGGAGGAGGTGGGGGA**TGG** | Csa4G088720 | 3 | none |
| Target2 | T2-off1 | TTCGGCCGCTATGATACAA**TGG** | Csa2G032750 | 5 | none |
|  | T2-off2 | GACCACCATTGTTGTGCAA **TGG** | Csa2G357200 | 5 | none |
|  | T2-off3 | GGCGGCGATTAAGGTGCAA**TGG** | Csa6G134890 | 5 | none |
|  | T2-off4 | TTCCGTTATTATGCGACAA**TGG** | Csa6G188690 | 5 | none |

Note: PAM sequences or potential PAM sequences are written in bold; Mismatch bases are written in red letters.

**Table S3. Analysis of the potential off-target sites for *CsCLV1* sgRNA.**

| Targets | Potential off-target sites | | | | Presenceof mutation |
| --- | --- | --- | --- | --- | --- |
|  | **Name** | **Sequence** | **Gene** | **Number of mismatches** |  |
| Target1 | T1-off1 | TTCATGGAAACAATCTTAA**CGG** | Csa5G014290 | 5 | none |
|  | T1-off2 | TTCGGTGAAACATGGTGAC**GGT** | Csa6G306340 | 4 | none |
| Target2 | T2-off1 | AAGCTCGACGGAAACTCTC**CGG** | Csa2G005900 | 3 | none |
|  | T2-off2 | AAAGTCGTCCGTATCTCTC**TGG** | Csa1G237060 | 3 | none |
|  | T2-off3 | AAACTCGCCGGAATCTCGC**CGG** | Csa1G075600 | 3 | none |

Note: PAM sequences or potential PAM sequences are written in bold; Mismatch bases are written in red letters.

**Table S4. Analysis of the potential off-target sites for *CsGPA1* sgRNA.**

| Targets | Potential off-target sites | | | | Presenceof mutation |
| --- | --- | --- | --- | --- | --- |
|  | **Name** | **Sequence** | **Gene** | **Number of mismatches** |  |
| Target1 | T1-off1 | AAGCTCATCAAACCCAATT**TGG** | Csa1G648050 | 4 | none |
|  | T1-off2 | TGCTCCATCAAAACAATTT**TGG** | Csa6G490970 | 5 | none |
|  | T1-off3 | TGCATCACCAAAACCAGTT**CTG** | Csa2G403730 | 4 | none |
| Target2 | T2-off1 | GGATACCCCGCGTTTGACT**TAA** | Csa3G603590 | 4 | none |

Note: PAM sequences or potential PAM sequences are written in bold; Mismatch bases are written in red letters.

**Table S5 Genes information used in this study.**

| **Gene name** | **Species** | **Accession** |
| --- | --- | --- |
| *AtGPA1* | *Arabidopsis thaliana* | NP_001325056 |
| *SlTGA1* | *Solanum lycopersicum* | NP_001292984 |
| *GmSGA1* | *Glycine max* | NP_001238184 |
| *OsRGA1* | *Oryza sativa L* | NP_001407289 |
| *CT2* | *Zea mays* | NP_001151085 |
| *TaGPA1* | *Triticum aestivum* | ADV39994 |
| *CsGPA1* | *Cucumis sativus* | CsaV3_4G036110 |
| *CsCLV1* | *Cucumis sativus* | CsaV3_3G045960 |
| *CsCLV3* | *Cucumis sativus* | CsaV3_1G015250 |
| *CsWUS* | *Cucumis sativus* | CsaV3_6G047050 |
| *CsCRC* | *Cucumis sativus* | CsaV3_5G033400 |
| *CsUBI* | *Cucumis sativus* | CsaV3_5G031430 |

**Table S6 Primers used in this study.**

| **Primers for qRT-PCR** | |
| --- | --- |
| *qCsWUS-F* | CTCTGCTAGGCTTAGACAGTAC |
| *qCsWUS-R* | TTCTGTCTTTCACGAGCCTTAT |
| *qCsCLV1-F*  *qCsCLV1-R*  *qCsCLV3-F*  *qCsCLV3-R*  *qCsGPA1-F*  *qCsGPA1-R*  *qCsCRC-F*  *qCsCRC-R* | GAATAACAACTTCACGCTCGAG  AGGCCAGTTAGATGATTAGTCG  ATGCTTGTAGAGATGAAGAAGGAG  TCAAGGAGTTCTAGGCTTCTTTGG  GCGATTCAGGAAACGTATTCTC  CTGGATTTCAACAACACCAGTT  CACTGTTCTTGCGGTTGGG  CTGAGACTGAAAAGTTAGAGGATG |
| *CsUBI-F* | CACCAAGCCCAAGAAGATC |
| *CsUBI-R* | TAAACCTAATCACCACCAGC |
| **Primers for in situ probes** | |
| *CsCLV1-SP6* | GATTTAGGTGACACTATAGAATGCTACGCTAGGGCGAATCAAACA |
| *CsCLV1-T7*  *CsGPA1-SP6*  *CsGPA1-T7*  *CsWUS-SP6*  *CsWUS-T7* | TGTAATACGACTCACTATAGGGGGTATTCTGTGAGGCGGGAG  GATTTAGGTGACACTATAGAATGCTCAGTTTAGCCCCGTTGGTGA  TGTAATACGACTCACTATAGGGTCAAAGAGATTTCGCCGCCT  GATTTAGGTGACACTATAGAATGCTTTCTCCTATTACTACCTCCAATACT TGTAATACGACTCACTATAGGGTGCAGAAACCACCGAGAT |
| **Primers for genetic transformation and gene amplification** | |
| *CsCLV1-BsF* | ATATATGGTCTCGATTGTTCGGGGAAACATGCTTACGTT |
| *CsCLV1-F0* | TGTTCGGGGAAACATGCTTACGTTTTAGAGCTAGAAATAGC |
| *CsCLV1-DT2-R0* | AACGAGAGATACCGTCGAGTTTCAATCTCTTAGTCGACTCTAC |
| *CsCLV1-DT2-BsR* | ATTATTGGTCTCGAAACGAGAGATACCGTCGAGTTTCAA |
| *CsGPA1-BsF* | ATATATGGTCTCGATTGTGCCTCATCAAACCCAGTTGTT |
| *CsGPA1-F0* | TGTGCCTCATCAAACCCAGTTGTTTTAGAGCTAGAAATAGC |
| *CsGPA1-DT2-R0* | AACAGTCAAACGCGGGTAATCCCAATCTCTTAGTCGACTCTAC |
| *CsGPA1-DT2-BsR* | ATTATTGGTCTCGAAACAGTCAAACGCGGGTAATCCCAA |
| *CsCLV3-BsF* | ATATATGGTCTCGATTGGGCGGGAAGAGGTGGGGGAGTT |
| *CsCLV3-F0* | TGGGCGGGAAGAGGTGGGGGAGTTTTAGAGCTAGAAATAGC |
| *CsCLV3-DT2-R0* | AACTTGCACCATAATGGCGGAACAATCTCTTAGTCGACTCTAC |
| *CsCLV3-DT2-BsR* | ATTATTGGTCTCGAAACTTGCACCATAATGGCGGAACAA |
| *CsCLV1-clone-mutant-F* | ACGGCTGAAATCACGGTAGAA |
| *CsCLV1-clone-mutant-R* | TTGCTGTTACGCCCGAGATTC |
| *CsGPA1-clone-mutant1-F* | ATGTGTGCTACACTGCTACTTAGT |
| *CsGPA1-clone-mutant1-R* | TGAGCAAGCTCCTTCGAACCA |
| *CsGPA1-clone-mutant2-F* | GAATACACACGAGCCTTATCCAGC |
| *CsGPA1-clone-mutant2-R* | CACTGATAGACTTGTATCGCCGAT |
| *CsCLV3-clone-mutant-F* | ATGCTTGTAGAGATGAAGAAGG |
| *CsCLV3-clone-mutant-R* | TCAAGGAGTTCTAGGCTTCTTTGG |
| **Primers for yeast two-hybrid, co-IP analysis, firefly luciferase complementation imaging (LCI) assay** | |
| *CsCLV1*_657-973_*-BD-F* | CATGGAGGCCGAATTCATGCGGAAGAGAAAGAAGATTCA |
| *CsCLV1*_657-973_*-BD-R* | GGATCCCCGGGAATTC TTAGAGATTGATTAGAGTAGGGGC |
| *CsGPA1-AD -F* | GGCCAGTGAATTCATGCTGTCTCATTTGAGTAGAAA |
| *CsGPA1-AD -R* | CCGGGTGGAATTCTCACAATAACCCAGCCTCAAAGA |
| *CsGPA1-BIFC-F* | CGCGCCACTAGTGGATCCATGCTGTCTCATTTGAGTAGAA |
| *CsGPA1-BIFC-R* | AGTACTATCGATGGATCCCAATAACCCAGCCTCAA |
| *CsCLV1-BIFC-F* | CGCGCCACTAGTGGATCCATGAAGAGAAGACCGATTGATCC |
| *CsCLV1-BIFC-R*  *CsGPA1-cLUC-F*  *CsGPA1-cLUC-R* | AGTACTATCGATGGATCCGAGATTGATTAGAGTAGGGGCA  TACGCGTCCCGGGGCGGTACCATGCTGTCTCATTTGAGTAGAA  ACGAAAGCTCTGCAGGTCGACTCACAATAACCCAGCCTCAA |
| *CsCLV1-nLUC-F* | CGGGGGACGAGCTCGGTACCATGAAGAGAAGACCGATTGATCC |
| *CsCLV1-nLUC-R* | ACGCGTACGAGATCTGGTCGACGAGATTGATTAGAGTAGGGGCA |
| *CsCLV1-cGFP-F* | GACTCTAGTCTAGAAAGCTTATGAAGAGAAGACCGATTGATCC |
| *CsCLV1-cGFP-R* | CCCTTGCTCACCATGGTACCGAGATTGATTAGAGTAGGGGCA |
| *CsGPA1-FLAG-F* | CAAATCGACTCTAGAAAGCTTATGCTGTCTCATTTGAGTAGAA |
| *CsGPA1-FLAG-R* | ATGGTACCGGATCCACTAGTCAATAACCCAGCCTCAA |
| **Primers for potential off-target sites analysis** | |
| *CsCLV3-T1-off1-F* | ATGGGTGAAGGCCGAAGG |
| *CsCLV3-T1-off1-R* | CTAGCGATCCTGACAAGTACGG |
| *CsCLV3-T1-off2-F* | AACATCGTCAAAGGAATTGGG |
| *CsCLV3-T1-off2-R* | GATTTCGTTGAGGATCCAGACT |
| *CsCLV3-T2-off1-F* | GACGCATTGCTGGAAACAAA |
| *CsCLV3-T2-off1-R*  *CsCLV3-T2-off2-F*  *CsCLV3-T2-off2-R*  *CsCLV3-T2-off3-F*  *CsCLV3-T2-off3-R*  *CsCLV3-T2-off4-F*  *CsCLV3-T2-off4-R*  *CsCLV1-T1-off1-F*  *CsCLV1-T1-off1-R*  *CsCLV1-T1-off2-F*  *CsCLV1-T1-off2-R*  *CsCLV1-T2-off1-F*  *CsCLV1-T2-off1-R*  *CsCLV1-T2-off2-F*  *CsCLV1-T2-off2-R*  *CsCLV1-T2-off3-F*  *CsCLV1-T2-off3-R*  *CsGPA1-T1-off1-F*  *CsGPA1-T1-off1-R*  *CsGPA1-T1-off2-F*  *CsGPA1-T1-off2-R*  *CsGPA1-T1-off3-F*  *CsGPA1-T1-off3-R*  *CsGPA1-T2-off1-F* | AAAGGGCACAGTGAACATCAT  GATCTGAAGCTCAATCCAGGA  GTATGCAGAACCAGGAGGG  AGAAACGTCAAGATTTCAGGAAT  CTAGAGTGTTTCACAAACCGC  GTTGTGGGTACAGGATTAATTGATA  CCAAAGCTTCCTCACCAATT  GCAATATGTCGAAGCTTAGCTAC  AATCATCTGAACACTTCGAAGATTC  ATGGATGGGAATAAGGACGAAG  TCAAACAACTGAAAGCCTTGG  TCTAATTCCGTCTGTAATTGGGT  GTCTCTAACAACAAACTCAACGG  GAAAAGAGAAAACCTCCCTCGA  ATTTAATACCCGCCATAATGTGC  GAACAAGTTGGAAGGGAAGTTAC  AATGAAAATGGAAAGGTTCTTAGCA  ATGATGAAGTCGCAAGGATATGT  ATTGCATTTGCTTATGTTGTTCC  GTCGGCCTTTGGCTTTATT  ACCACATTACTTGCTTTATACCCT  GGCCAAAAGTTGCATCATTC  CATTGATGATGGACTACGAACG  ATGCTTCATAATCGTCTCCTCG |
| *CsGPA1-T2-off1-R* CTCGACGACAAAATCTCATTCAC | |
